# GPER1 reduces skin inflammation by inhibiting keratinocyte proliferation

**DOI:** 10.1038/s41420-026-03059-1

**Published:** 2026-03-21

**Authors:** Natalia Pérez-Escudero, Isabel Cabas, Raúl Corbalán-Vélez, Teresa Martínez-Menchón, Belén Ferri, María L. Cayuela, Diana García-Moreno, Alfonsa García-Ayala, Victoriano Mulero

**Affiliations:** 1 Departmento de Biología Celular e Histología, Facultad de Biología, Universidad de Murcia, Murcia, Spain; 2 Instituto Murciano de Investigación Biosanitaria (IMIB)-Pascual Parrilla, Murcia, Spain; 3 Centro de Investigación Biomédica en Red de Enfermedades Raras (CIBERER), Instituto de Salud Carlos III, Madrid, Spain; 4 Hospital Clínico Universitario Virgen de la Arrixaca, Murcia, Spain

**Keywords:** Chronic inflammation, Cell death and immune response, Psoriasis

## Abstract

Psoriasis is a chronic inflammatory skin disease characterized by keratinocyte (KC) hyperproliferation and immune cell infiltration, including neutrophils. While estrogens are known to modulate immune responses, the role of the G protein-coupled estrogen receptor 1 (GPER1) in skin inflammation remains poorly understood. Here, we show that GPER1 signaling is downregulated in lesional skin of psoriasis patients and negatively correlates with both inflammation markers and KC proliferation. Using a zebrafish model of chronic skin inflammation (Spint1a-deficient larvae), we demonstrate that Gper1 deficiency leads to increased KC proliferation and enhanced neutrophil infiltration, without directly modulating inflammatory signaling. Pharmacological inhibition of cell proliferation with palbociclib reduced both KC aggregates and neutrophil infiltration, independently of NF-κB activation. Moreover, Gper1 overexpression in basal KCs, but not in neutrophils, rescued skin alterations, indicating a cell-autonomous effect in KCs. Notably, our results also suggest that epithelial cell proliferation facilitates immune cell infiltration into inflamed tissue. Together, our results identify GPER1 as a negative regulator of keratinocyte hyperproliferation and skin inflammation, suggesting that modulation of this pathway may represent a therapeutic strategy for hyperproliferative inflammatory skin diseases such as psoriasis.

## Introduction

Multiple lines of evidence indicate that genetic, epigenetic and environmental factors are involved in the progression of several immune disorders. One prominent example is psoriasis, a chronic autoimmune inflammatory skin disease characterized by inflamed, scaly plaques, often associated with disfigurement, disability, and comorbidities [1, 2], and high prevalence (2–3% and affecting around 125 million people worldwide). This disorder involves complex pathogenic interactions between the innate and adaptive immune system, being a T cell–mediated disease primarily driven by pathogenic T cells that produce high levels of interleukin-17 (IL-17) in response to IL-23 [2, 3]. Psoriasis pathogenesis is characterized by epidermal hyperplasia due to excessive keratinocyte (KC) proliferation, activation of the innate immune response, and infiltration of immune cells—particularly neutrophils—into the epidermis. These events are accompanied by the release of proinflammatory cytokines such as IL-36, IL-1β, CXCL8 and interferon γ (IFNγ), which act synergistically with tumor necrosis factor α (TNFα) to sustain chronic skin inflammation [2, 3].

Added to that complexity, there is great evidence for a sex bias in the incidence of several immune disorders [4, 5], including psoriasis. Although the mechanisms underlying these sex-related differences are not fully understood, the presence of sex hormone receptors—such as estrogen receptors—on immune cells is thought to contribute to their immunomodulatory effects. Beyond their essential role in reproductive biology, estrogens participate in a wide range of physiological processes across various tissues, including in males, and are known to modulate several aspects of immune function [6]. Estrogen actions are mediated through classical nuclear estrogen receptors (ERs), which mediated the classical genomic pathway (signals over hours or days), but also by membrane ERs, such G protein-coupled estrogen receptor 1 (GPER1), which mediates nongenomic responses (signals even within seconds) through activating several signaling pathways, such as RAS/MEK/ERK, PI3K/AKT, AC/cAMP/PKA/CREB, and PLC/IP3/CAMK [7]. Both receptor subtypes have been described to be expressed in a wide variety of immune cell types, including cells orchestrating inflammatory responses. Since its identification in 2005, studies using GPER1-deficient mice and the selective agonist G-1 have shown that GPER1 regulates numerous physiological processes and is implicated in a wide range of diseases, including cardiovascular, renal, metabolic, gastrointestinal, hepatic, and neurological disorders, as well as in physiological ageing and autoimmunity [8–10]. Although its functions are not yet fully elucidated, increasing evidence supports a role for GPER1 in immune-mediated conditions such as multiple sclerosis, Parkinson’s disease, inflammatory bowel disease, and atherosclerosis-associated inflammation[11]. Notably, GPER1 deletion has been linked to a form of familial immunodeficiency [11]. In addition, GPER1 activation has been shown to reduce dermonecrosis and enhance bacterial clearance in a mouse model of *Staphylococcus aureus* skin infection [12] and to modulate skin inflammation triggered by lupus IgG in a systemic lupus erythematosus model [13].

As regards psoriasis, it has been reported that incidence and severity are higher in men than in women [14, 15], and its severity decreases during pregnancy, when estrogen levels are higher [16]. Thus, the severity of psoriasis has been described to inversely correlate with serum estrogen levels. However, other reports failed to observe these differences [17]. Estrogens have been reported to modulate KC activation as well as IL-1β production by neutrophils and macrophages[17, 18]. However, their immunomodulatory effects in psoriasis remain controversial, as both pro- and anti-inflammatory actions have been described [19]. In addition, women with moderate to severe psoriasis often show better responses to phototherapy than men [20], although the underlying mechanisms remain unclear. To date, most evidence regarding the role of estrogens in psoriasis arises from epidemiological and in vitro studies, highlighting the need for further investigation into their function in psoriatic inflammation in vivo. In addition, while these effects have primarily been attributed to nuclear ERs, the contribution of GPER1 in this context remains unexplored.

In this study, we utilized human biopsies and transcriptional datasets from psoriasis patients to demonstrate that GPER1 signaling is suppressed in psoriatic conditions. Furthermore, employing the Spint1a-deficient mutant zebrafish line—an established model of chronic skin inflammation that mirrors key features of the psoriasis model [21]—we discovered that Gper1 exerts a protective role in skin inflammation by specifically regulating KC proliferation, without directly influencing the inflammatory response. Importantly, our findings reveal that KC proliferation is essential for facilitating neutrophil infiltration into inflamed skin.

## Results

### GPER1 transcript levels negatively correlate with disease severity in lesional skin from psoriasis patients

**Fig. 1:**
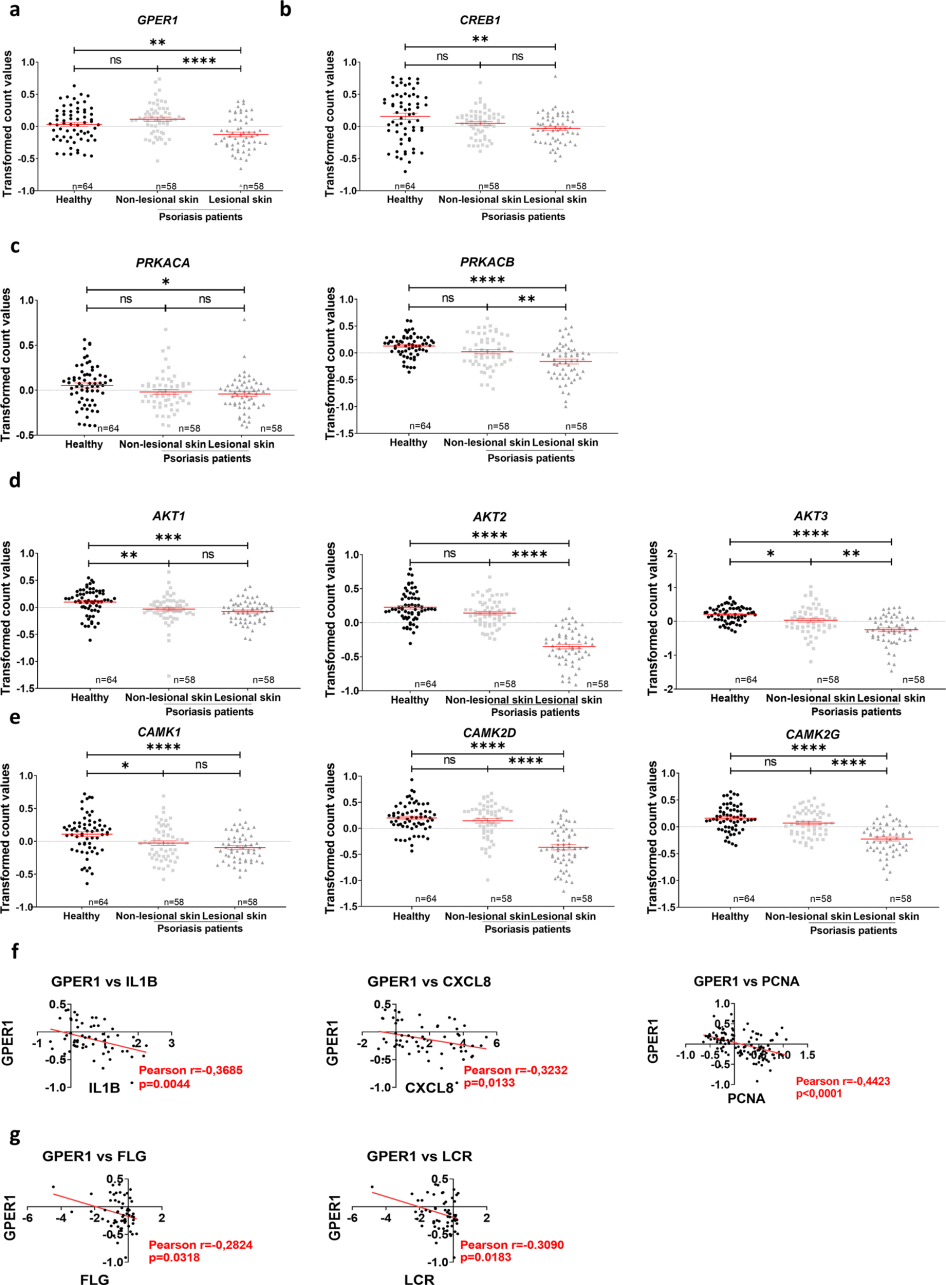
Differential expression profile of the genes related to the GPER1 signaling pathway in psoriasis lesional skin compared to healthy or non-lesional skin. Transcriptomic data obtained from a human psoriasis patient cohort (GSD4602) from the GEO database. **a**–**e** Comparison of the expression of several genes implicated in the analyzed pathway between healthy, non-lesional and lesional skin. **f**, **g** Analysis of the correlation between *GPER1* and the inflammatory markers *IL1B* and *CXCL8*, the proliferation marker *PCNA*, and the differentiation markers *FLG* and *LRC*. Each point represents one individual, and the mean ± SEM for each group is also shown. *p-*values were calculated using one-way ANOVA in Graphs (**a**–**e**) and Pearson Correlation test in Graph (**f**). * *p* ≤ 0.05, ** *p* ≤ 0.01, *** *p* ≤ 0.001, **** *p* ≤ 0.0001.

To ascertain if GPER1 plays a significant role in psoriasis, a detailed study of the expression of several genes related to the GPER1 signaling pathway was performed. We analyzed the transcriptomic data from a dataset of human psoriasis patients contained in the GEO database GSD4602, which included data from 180 samples of non-lesional and lesional skin of psoriasis patients compared to skin of healthy subjects. The analysis revealed a differential expression profile of the studied genes in psoriasis-lesional skin compared to healthy or non-lesional skin. In psoriatic lesional skin, we observed lower transcript levels of *GPER1* (Fig. 1a) and *CREB1*, which encodes the transcription factor cAMP responsive element binding protein (Fig. 1b). Notably, the transcript levels of *PRKACA* and *PRKACB*, which encode the α and β catalytic subunits of protein kinase A (PKA), respectively, were significantly reduced in lesional skin compared to non-lesional or healthy skin (Fig. 1c). Furthermore, *AKT1*, *AKT2*, and *AKT3*, which encode the 3 isoforms of PKB, and *CAMK1*, *CAMK2D* and CAMK2G, which encode calcium^/^calmodulin-dependent protein kinase family members, also showed lower mRNA levels in psoriasis lesional skin than in non-lesional and healthy skin (Fig. 1d, e). Notably, the data also revealed a negative correlation between the expression of *GPER1* and those of inflammatory (*IL1B* and *CXCL8*), proliferation (proliferating cell nuclear antigen, *PCNA*), and differentiation (filaggrin, *FLG*, and loricrin, *LRC*) biomarkers (Fig. 1f, g).

**Fig. 2:**
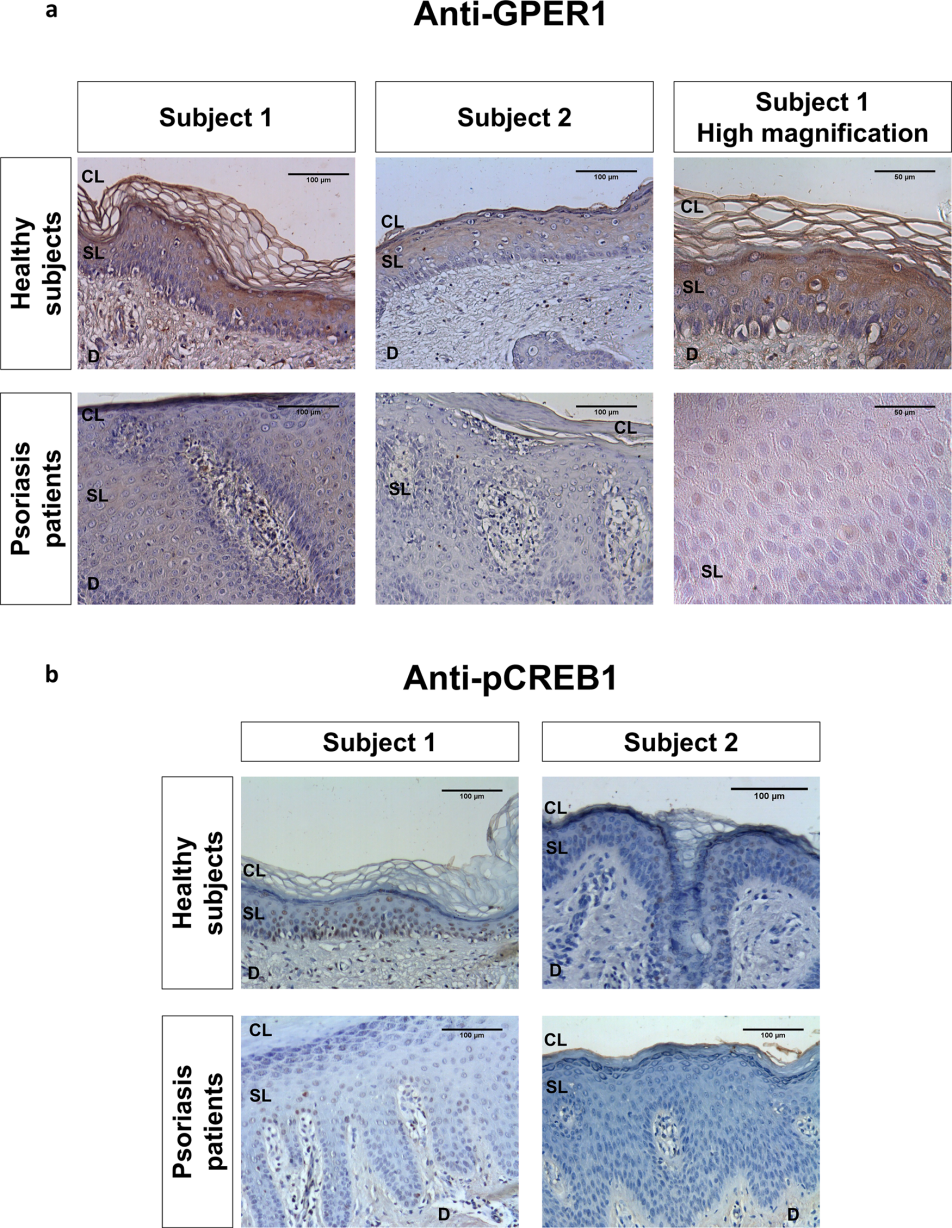
Psoriasis lesional skin has lower protein levels of GPER1 and the activated form of CREB. Representative images of biopsy sections from healthy and psoriasis patients immunostained with anti-GPER1 (**a**) and anti-pCREB (**b**). Sections were examined under a Leica microscope DMC6200 equipped with a Leica DFC 280 digital camera. No staining was observed when primary antibodies were omitted. Magnification at 20X and 40X (scale bar 100 µm and 50 µm, respectively). CL Cornified layer, SL Spinous layer, D dermis.

To determine if the alterations of transcript levels of genes involved in GPER1 signaling indeed resulted in altered signaling, we detected GPER1 and pCREB1 (the active form of CREB1 that is phosphorylated at S133) by immunohistochemistry in lesional skin from psoriasis patient biopsies. The results showed lower expression of GPER1 in skin biopsies of psoriasis patients than in healthy skin (Fig. 2a). In addition, GPER1 showed a cytoplasmic distribution in healthy skin, while it was also located in the KC nuclei of psoriatic skin (Fig. 2a). Moreover, pCREB1 immunoreactivity was found in the nuclei of KC of basal and spinous layers of healthy skin, whereas they were hardly detected in psoriatic lesional skin, probably because of the lower expression of GPER1 in psoriasis (Fig. 2b). Collectively, these results suggest that the GPER1 signaling pathway may have a protective role in psoriasis.

### Gper1 deficiency promotes keratinocyte aggregation and neutrophil infiltration without directly modulating inflammation in a zebrafish model of psoriasis

**Fig. 3:**
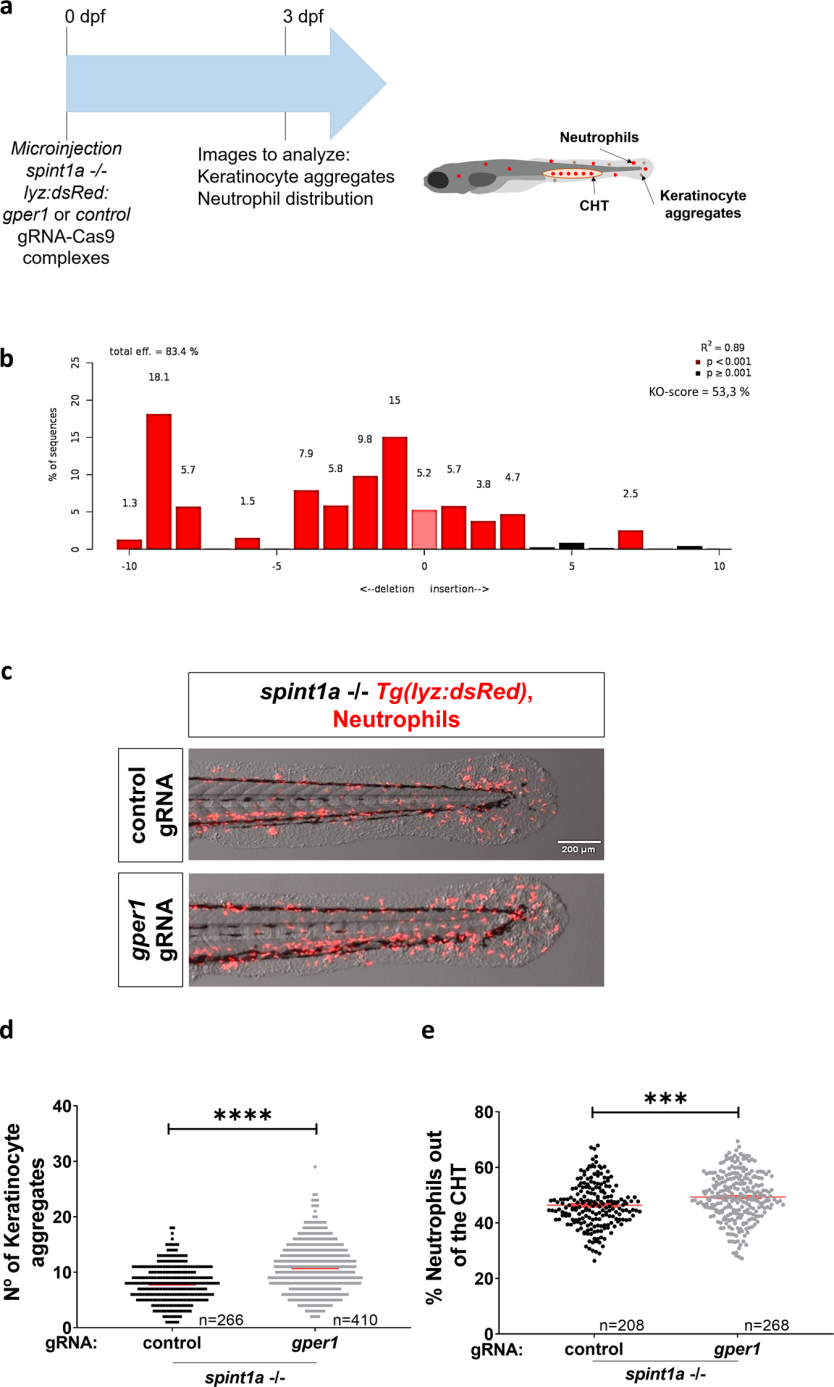
Genetic inhibition of *gper1* aggravates skin alterations and neutrophil infiltration in Spint1a-deficient larvae. **a** Schematic of the experimental procedure used. Eggs at one-cell stage were microinjected with CRISPR-Cas9 system, using *gper1* or control gRNA. At 3 dpf, images were taken to analyze KC aggregates and neutrophil distribution. **b** Analysis of genome editing efficiency in larvae injected with *gper1* gRNA/Cas9 complexes and quantification rate of nonhomologous end joining mediated repair, showing all insertions and deletions at the target site using TIDE (https://tide.nki.nl). **c** Representative images of 3 dpf control and Gper1-deficient larvae. **d** Quantification of the number of skin KC aggregates in control and Gper1-deficient larvae. **e** Quantification of neutrophil distribution in the tail in control and Gper1-deficient larvae. Each dot represents one individual, and the means and SEM for each group are also shown. *P* values were calculated by Student’s *t* test. ***P ≤ 0.001; ****P ≤ 0.0001.

**Fig. 4:**
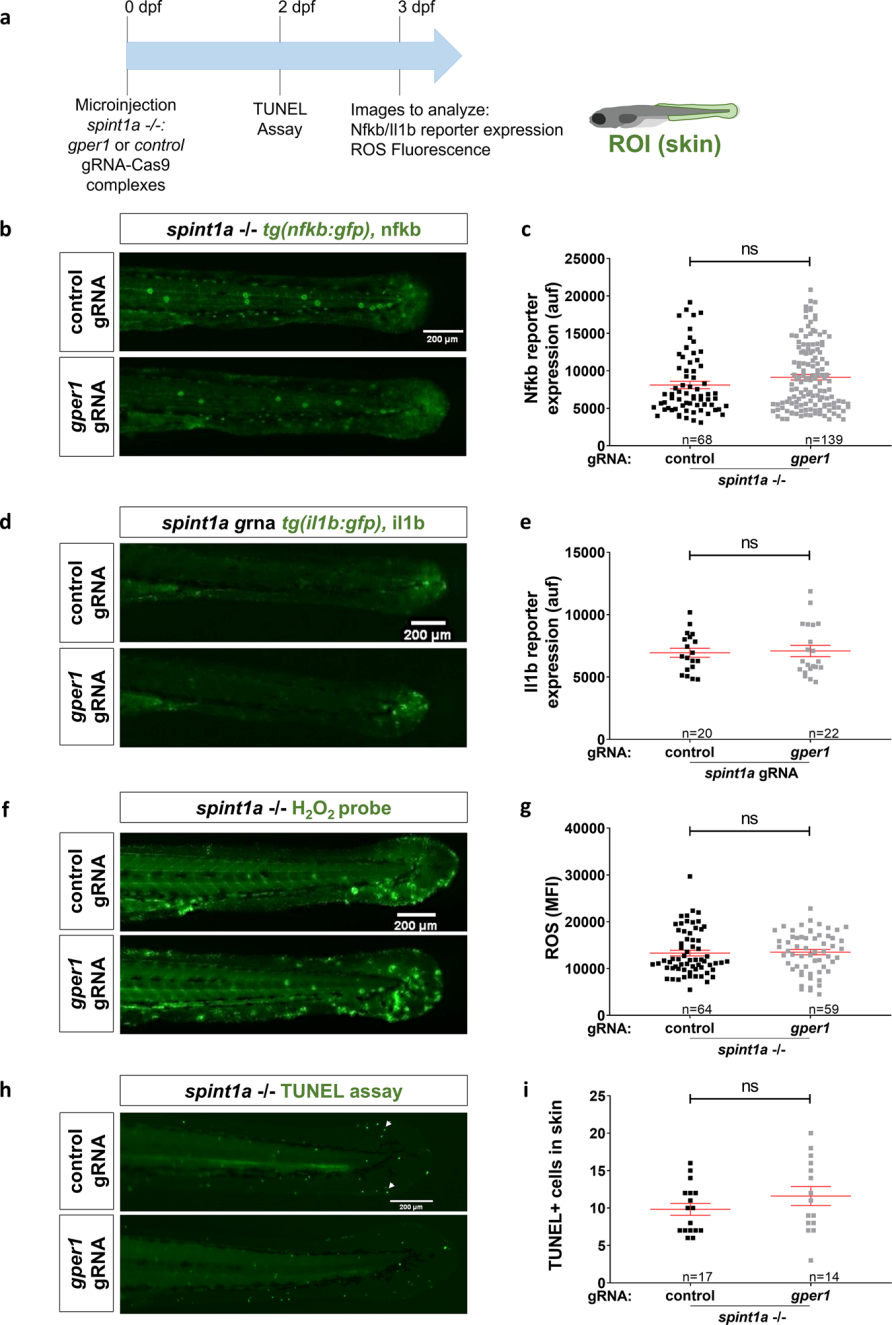
Genetic inhibition of *gper1* did not affect inflammatory markers in Spin1a-deficient larvae. **a** Schematic of the experimental procedure used. Spint1a-deficient one-cell stage eggs were microinjected with CRISPR-Cas9 system, using *gper1* or control gRNA. At 2 dpf, TUNEL assay was performed. At 3 dpf, images were taken to analyze Nfkb/Il1b reporter expression or ROS fluorescence. **b** Representative images of 3 dpf control and Gper1-deficient NFkB:GFP+ larvae. **c** Quantification of Nfkb activity in skin in each group. **d** Representative images of 3 dpf control and Gper1-deficient il1b:GFP+ larvae. **e** Quantification of Il1b expression in skin in every condition. **f** Representative images of 3 dpf control and Gper1-deficient larvae, incubated with H_2_O_2_ fluorescent probe. **g** Quantification of skin H_2_O_2_ production in every group. **h** Representative images of 2 dpf control and Gper1-deficient larvae. **i** Number of TUNEL+ cells in skin in each condition. Each dot represents one individual, and the means and SEM for each group are also shown. *P* values were calculated by Student’s *t* test when parametric (Graphs **c**, **g**, **i**) or Mann–Whitney test when no parametric (Graph **e**). ns, not significant.

To confirm the putative protective role of GPER1 in psoriasis, we next inactivated Gper1 by CRISPR-Cas9 in Spint1a-deficient zebrafish larvae which are an excellent model of chronic skin inflammation (Fig. 3a). It was found that Gper1-deficient larvae (KO efficiency of about 50%) (Fig. 3B) exhibited slightly higher number of KC aggregates (Fig. 3c, d) and increased neutrophil skin infiltration (Fig. 3c, e). These results prompted us to investigate the mechanism by which this receptor acts in this inflammatory context. Initially, we wondered if the genetic inhibition of *gper1* could have a direct impact on inflammation by analyzing Nfkb activation and Il1b production using *nfkb:eGFP* and *il1b: GFP* zebrafish reporter lines, respectively (Fig. 4a). Unexpectedly, no significant differences were observed in any of these inflammation markers between Gper1-deficient and their wild-type sibling larvae (Fig. 4b, e). Similarly, despite the critical role of oxidative stress and cell death in promoting skin inflammation in the Spint1a-deficient model [21], skin H_2_O_2_ production (Fig. 4f, g) and the number of TUNEL^+^ cells (Fig. 4h, i) were both unaffected by Gper1 deficiency. Taken together, these results indicate that although Gper1 deficiency increased the number of KC aggregates and promoted skin neutrophil infiltration, it did not directly regulate inflammation.

### The protective role of Gper1 is specific to its function in regulating keratinocyte proliferation

**Fig. 5:**
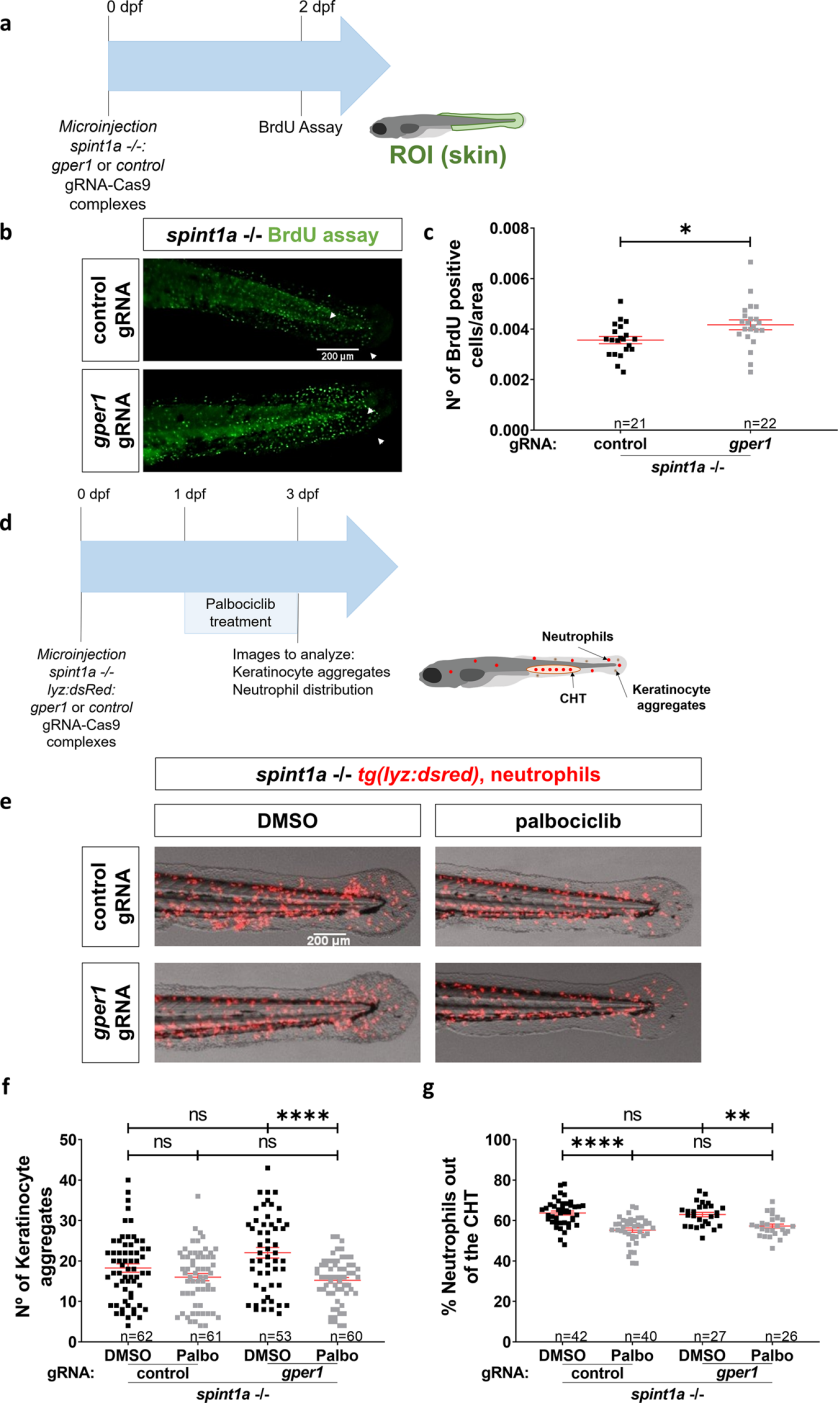
Gper1 role in chronic skin inflammation is related to the regulation of keratinocyte proliferation. **a** Schematic of the experimental procedure used. Eggs at one-cell stage were microinjected with CRISPR-Cas9 system, using *gper1* or control gRNA. At 2 dpf, a BrdU assay was performed. **b** Representative images of BrdU assay in 2 dpf control and Gper1-deficient larvae. **c** Quantification of BrdU+ cells per area in skin in both conditions. **d** Schematic of the experimental procedure used. Spint1a-deficient one-cell stage eggs were microinjected with the CRISPR-Cas9 system, using *gper1* or control gRNA. Treatments of interest were added to dechorionated embryos at 1 dpf by bath immersion and renewed daily. At 3 dpf, images were taken to analyze keratinocyte aggregates and neutrophil distribution. **e** Representative images of 3 dpf control and Gper1-deficient larvae, treated with Palbociblib (Palbo) or vehicle (DMSO). **f** Comparison of the number of KC aggregates in tail skin between non-edited and Gper1-deficient larvae, control or treated. **g** Quantification of neutrophil distribution in the tail in each group. Each dot represents one individual, and the means and SEM for each group are also shown. *P* values were calculated by Student’s *t* test in graphs (**b**, **d**) and by One-way ANOVA in graphs (**f**, **g**). ns not significant; *P ≤ 0.05; **P ≤ 0.01; ****P ≤ 0.0001.

**Fig. 6:**
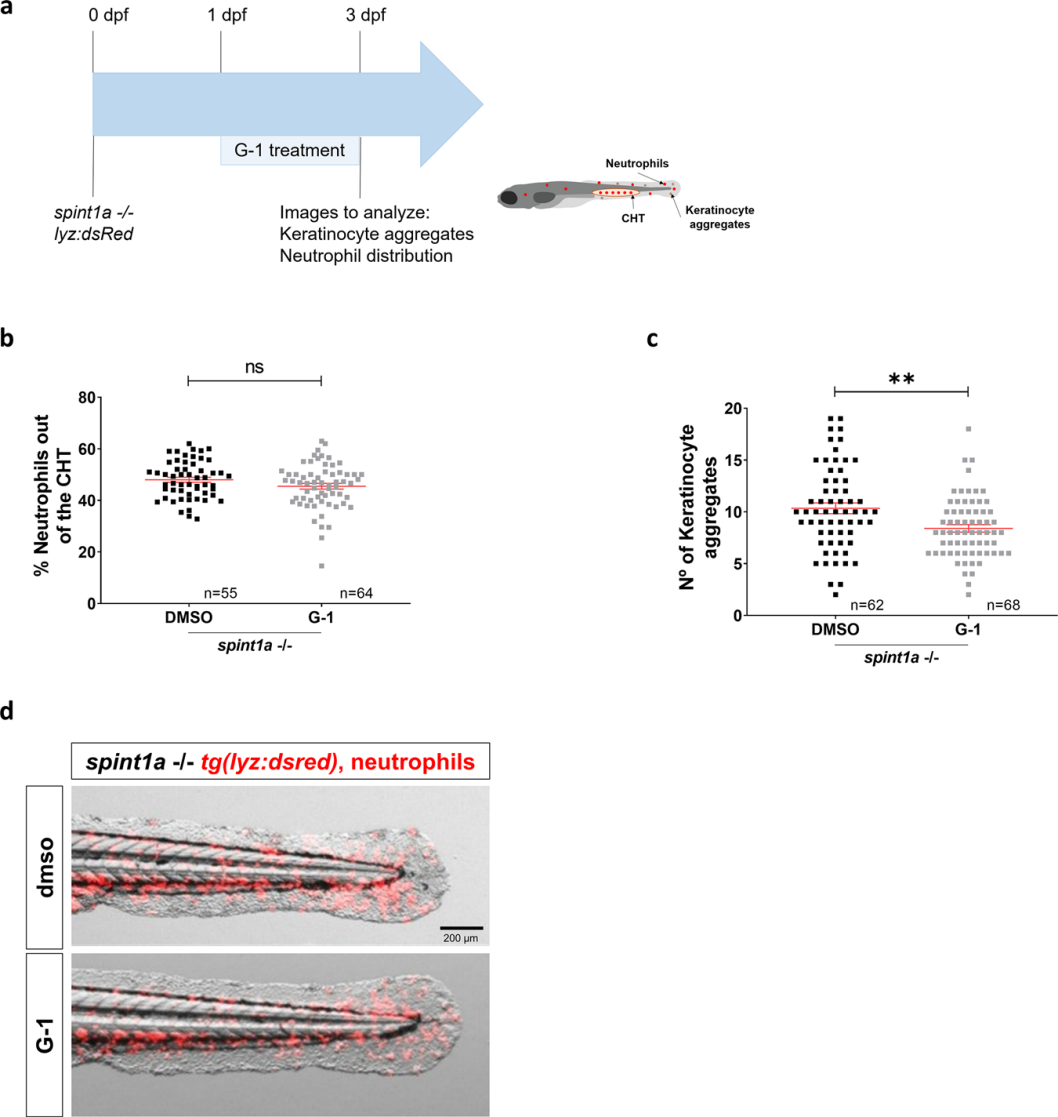
Pharmacological activation of Gper1 attenuates keratinocyte hyperproliferation in Spint1a-deficient larvae. **a** Schematic of the experimental procedure used. Spint1a-deficient embryos were treated with G-1 or vehicle (DMSO) at 1 dpf by bath immersion. At 2 dpf, images were taken to analyze neutrophil distribution and KC aggregates. Quantification of neutrophil distribution (**b**) and number of KC aggregates (**c**) in tail in every condition. **d** Representative merge (red and bright field) images of 3 dpf control and treated larvae, showing neutrophils and KC aggregates. Each dot represents one individual, and the means and SEM for each group are also shown. *P* values were calculated by Student’s *t* test (**c**, **d**, **f**) and by Pearson Correlation test (**g**). ns not significant; ***P ≤ 0.001.

Since Gper1 inhibition resulted in increased KC aggregate number and neutrophil infiltration in Spinta-deficient zebrafish larvae, but inflammation was unaffected, we wondered whether Gper1 was able to inhibit KC hyperproliferation, which is characteristic of this zebrafish model [22, 23] and psoriasis [3] (Fig. 5a). The results showed that Gper1 deficiency resulted in increased number of BrdU^+^ KC in Spint1a-deficient larvae (Fig. 5b, c), demonstrating not only that Gper1 signaling inhibited KC hyperproliferation but also that hyperproliferation promoted neutrophil infiltration. To further confirm the relevance of KC hyperproliferation on neutrophil infiltration, larvae were treated from either 1-3 dpf or 2-3 dpf with 10 μM palbociclib, a CDK4/CDK6 inhibitor approved for the treatment of certain types of breast cancer (Fig. 5d). It was found that the drug significantly reduced the number of KC aggregates, skin neutrophil infiltration and neutrophilia of Spint1a-deficient larvae (Fig. 5e–g). To further validate the role of Gper1 signaling, we treated Spint1a-deficient larvae with the selective Gper1 agonist G-1 (Fig. 6a). Treatment with 10 nM G-1 had no detectable effects, whereas 100 nM significantly reduced the number of KC aggregates without altering neutrophil infiltration (Fig. 6b–d), suggesting a specific effect on epithelial proliferation. Unfortunately, higher concentrations of G-1 (0.5 and 1 µM), which might have elicited stronger responses, induced developmental malformations (data not shown), preventing their use in the context of skin inflammation. These findings support that Gper1 signaling limits hyperproliferation of KC, thereby attenuating epithelial alterations, although its pharmacological activation is constrained by developmental toxicity at higher doses.

**Fig. 7:**
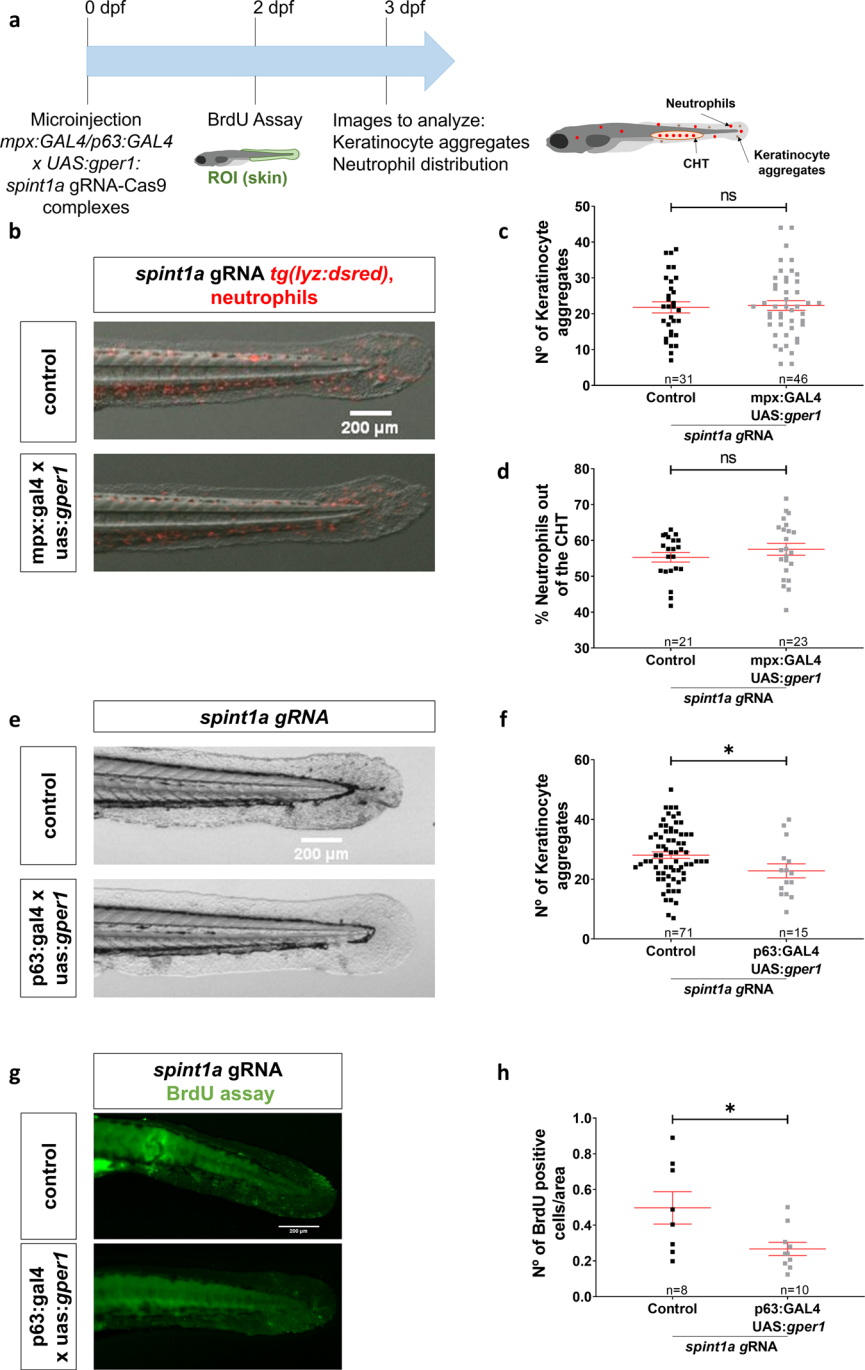
The role of gper1 in chronic skin inflammation is tissue-specific, as its overexpression in basal keratinocytes improves skin condition. **a** Schematic of the experimental procedure used. Zebrafish lines of interest were crossed to obtain eggs overexpressing *gper1* in either neutrophils or basal keratinocytes. A control group without overexpression was also obtained. At 2 dpf, proliferation was analyzed by BrdU assay. At 3 dpf, images were taken to analyze KC aggregates and neutrophils distribution. **b** Representative images of 3 dpf control larvae and larvae with *gper1*-overexpressing neutrophils. **c** Quantification of KC aggregates number in skin in both groups. **d** Quantification of neutrophil distribution in the tail in every condition. **e** Representative images of 3 dpf control larvae and larvae with *gper1*-overexpressing basal KC. **f** Number of KC aggregates in skin in every group. **g** Representative images of BrdU assay in 2 dpf control larvae and larvae with *gper1*-overexpressing basal KC. **h** Number of BrdU+ cells per area in skin in every group. Each dot represents one individual, and the means and SEM for each group are also shown. *P* values were calculated by Student’s *t* test. ns not significant; *P ≤ 0.05.

To further confirm the cell-autonomous effect of Gper1 signaling in KC, Gper1 was overexpressed in different cell types using the Gal4-UAS system [24] (Fig. 7a). While overexpression of Gper1 in neutrophils had no effects in the skin of Spint1a-deficient larvae, assayed as the number of KC aggregates and neutrophil infiltration (Fig. 7b–d), its overexpression in basal KC resulted in reduced number of KC aggregates (Fig. 7e, f) and BrdU^+^ KC (Fig. 7g, h). Collectively, these findings suggest that the exacerbation of the skin phenotype in Gper1-deficient larvae may be driven primarily by enhanced KC proliferation and that Gper1 mitigates skin inflammation indirectly by inhibiting KC hyperproliferation in a cell-autonomous manner.

### Keratinocyte proliferation is essential for facilitating neutrophil infiltration into inflamed skin

**Fig. 8:**
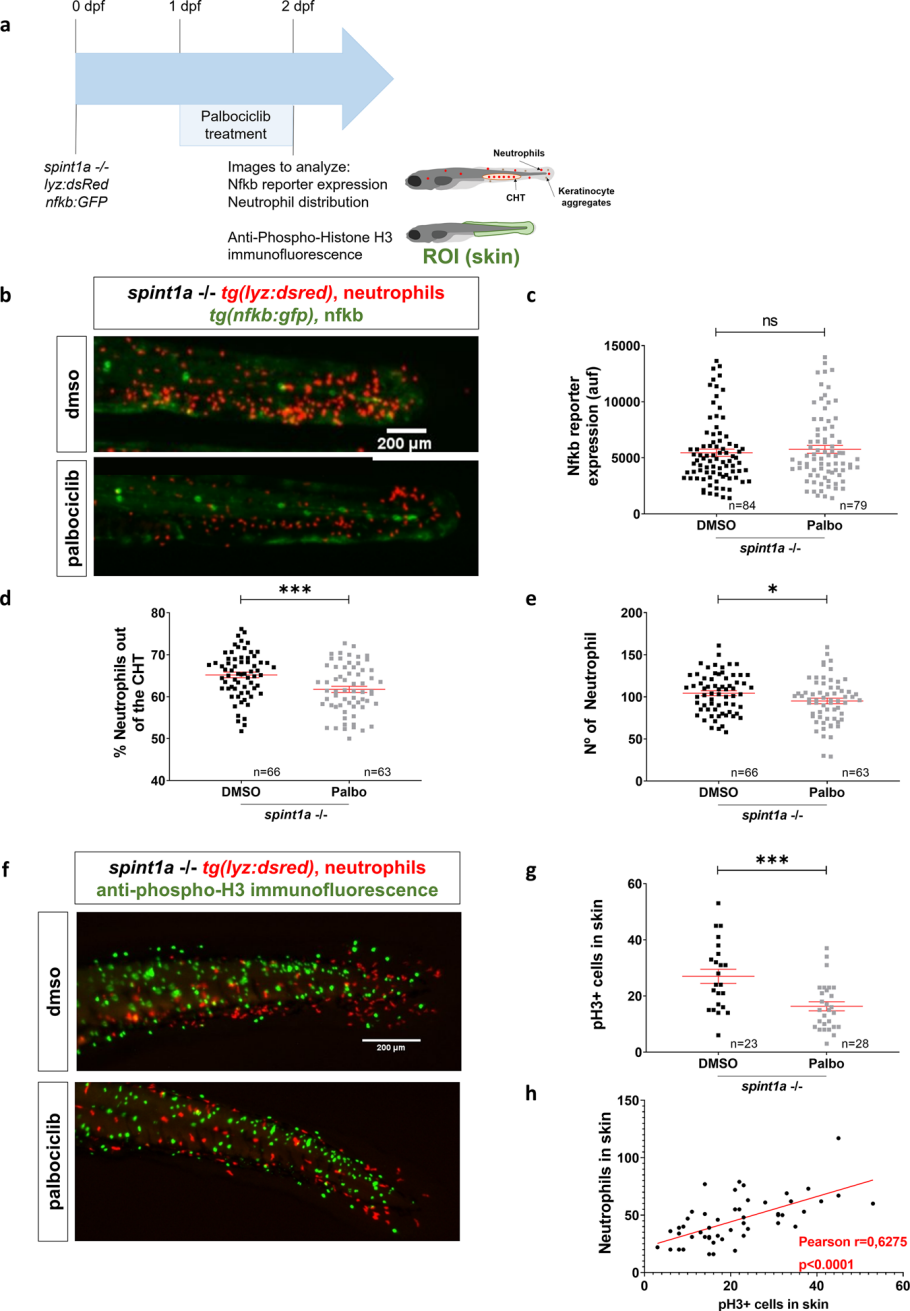
Keratinocyte proliferation is essential for facilitating neutrophil infiltration into inflamed skin. **a** Schematic of the experimental procedure used. Spint1a-deficient embryos were treated with Palbociclib or vehicle (DMSO) at 1 dpf by bath immersion. At 2 dpf, images were taken to analyze Nfkb reporter expression and neutrophil distribution. Anti-pH3 immunofluorescence was also performed. **b** Representative images of 2 dpf control and treated larvae, showing neutrophils and Nfkb reporter activity. **c** Quantification of Nfkb activity in skin in each group. Quantification of distribution (**d**) and number (**e**) of neutrophils in tail in every condition. **e** Quantification of neutrophil distribution in the tail in every condition. **f** Representative images of anti-pH3 immunofluorescence of 2 dpf control and treated larvae. **g** Number of pH3^+^ cells in skin in every condition. **h** Analysis of the correlation between the number of neutrophils and the number of pH3^+^ cells in skin. Each dot represents one individual, and the means and SEM for each group are also shown. *P* values were calculated by Student’s *t* test (**c**, **d**, **f**) and by Pearson Correlation test (**g**). ns, not significant; *P ≤ 0.05 ; ***P ≤ 0.001.

Given that Gper1 regulates keratinocyte proliferation in a cell-autonomous manner, we investigated whether changes in this proliferation also influenced neutrophil infiltration into the skin. To assess whether proliferation inhibition affected inflammation, we measured Nfkb activity (Fig. 8a). Despite a marked reduction in neutrophil skin infiltration, Nfkb activity remained unchanged in Spint1a-deficient larvae treated with Palbociclib (Fig. 8b–d). A slight reduction in the total number of neutrophils was also observed following palbociclib treatment (Fig. 8e); however, this is unlikely to account for the reduced skin infiltration, as infiltration was estimated by calculating the percentage of neutrophils located outside the caudal hematopoietic tissue (CHT)-the main residence of neutrophils in non-inflamed control larvae. These findings suggest that the effect of keratinocyte proliferation on neutrophil infiltration is independent of canonical inflammatory signaling.

**Fig. 9:**
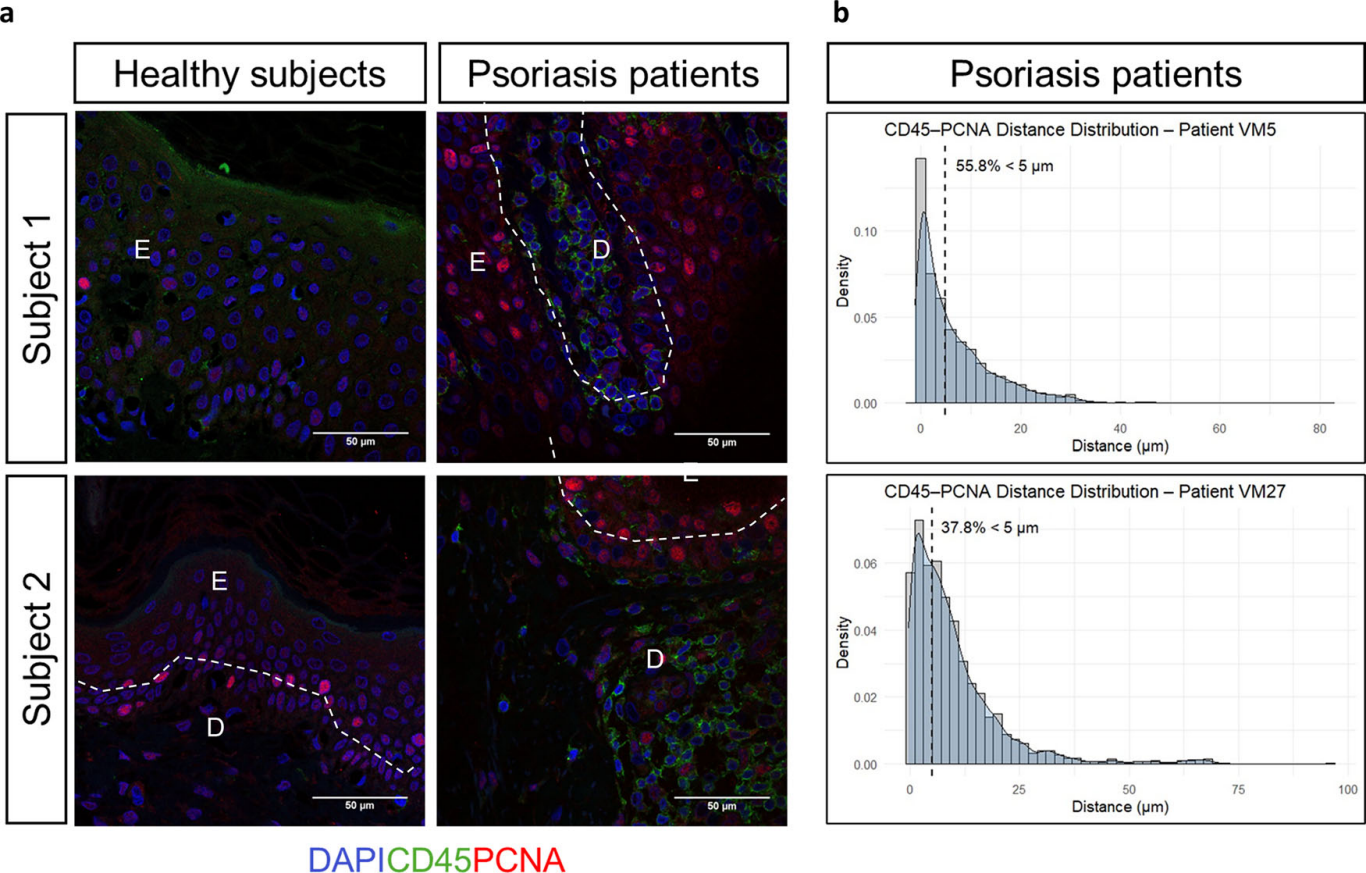
Leukocytes infiltrating the epidermis of psoriasis-lesional skin are located in close proximity to proliferating keratinocytes. **a** Representative images of skin biopsies from healthy subjects (HS) and psoriasis (PS) patients immunostained with a rabbit anti-human CD45 and a mouse anti-PCNA antibody, followed by the secondary antibodies donkey anti-rabbit IgG H&L Alexa Fluor® 488 and goat anti-mouse IgG H&L Alexa Fluor® 594. Sections were counterstained with DAPI and examined under a confocal microscope, Leica STELLARIS 8, and further processed with Leica software and ImageJ. The objective used was x63. Magnification at 63X (scale bar 50 µm). E epidermis, D dermis. **b** Analysis of the nearest neighbor assessing the Shortest Distance to Surface by Imaris; from surface 1 (green; CD45) to surface 2 (red; PCNA).

Since the relation between proliferation and infiltration of macrophages into epithelial tissues has already been described in the fruit fly embryo [25], we hypothesized that this mechanism might also contribute to neutrophil infiltration in the context of chronic skin inflammation. The results showed that Palbociclib reduced the number of pH3^+^ KC and, furthermore, revealed a positive correlation between the number of proliferative cells and the number of neutrophils in the inflamed skin (Fig. 8e–g). Strikingly, quantitation of distance distribution of infiltrated CD45^+^ cells and PCNA^+^ KC in the epidermis of two psoriasis patients revealed that 37.8 and 55.8% of the recorded events have a distance less than 5 μM, reflecting a high proximity between intraepithelial leukocytes and proliferating KC (Fig. 9a, b). As expected, no CD45^+^ cells were observed in the epidermis from healthy subjects, where proliferating KC were hardly observed (Fig. 9a). Collectively, these results suggest that KC proliferation is a key driver for neutrophil infiltration in inflamed skin.

## Discussion

Like other inflammatory disorders, psoriasis has a complex etiology, involving interactions between genetic, immune system, and multiple environmental factors. Although its complete pathogenesis is still not completely known, excessive KC proliferation, epidermal recruitment of leukocyte subsets (e.g., neutrophils) and production of multiple proinflammatory genes (e.g., IL-1β, IL-8, IL-17, IL-23, IL-36 and TNFα) are hallmarks of the disease [2, 3]. It is also widely accepted that estrogen has an immunomodulatory role [6], although its effects are sometimes controversial and depend on the concentration, type of target cell and the receptor subtype involved [17]. Moreover, to the best of our knowledge, no previous data showing the impact of GPER1 on psoriasis has been reported. However, GPER1 has been shown to play an important role in the pathogenesis of skin inflammation induced by lupus IgG [13] and in patients with epithelial permeability barrier dysfunction, including those with atopic dermatitis [12].

In the present study, we found evidence of the relevance of GPER1 signaling in psoriasis using patient samples. Thus, the transcript levels of *GPER1* and several markers of its signaling pathway, including *CREB1*, *PRKACA/B*, *AKT1-3* and *CAM1K/2KD/2KG*, were significantly reduced in lesional skin of psoriasis patients. In addition, consistent with this anti-inflammatory role of GPER1, a significant negative correlation between the expression of *GPER1* and the genes encoding the inflammatory cytokines IL-1β and CXCL8 was found in lesional skin from psoriasis patients. Moreover, *GPER1* expression also negatively correlated with the proliferation marker *PCNA* and with the differentiation markers *FLG* and *LRC*, supporting its role in modulating both KC hyperproliferation and impaired epidermal differentiation in psoriatic skin. The lower GPER1 and pCREB1 amounts in psoriasis lesional skin further confirmed the inhibition of GPER1 signaling pathway in this disease. Curiously, we have also observed altered localization of GPER1 in psoriasis lesional skin, and this may be relevant, since GPER1 is not always localized in the plasma membrane [26]. Thus, depending on the context, it can be endocytosed to reach the trans-Golgi apparatus and the endoplasmic reticulum [27], and it can even be translocated to the nucleus, possibly through importins, where it increases the expression of the oncogene *FOS* [28]. Moreover, GPER1 localization has been associated with distinct tumor properties of breast cancer [29]. Therefore, it is worth studying whether the localization of GPER1 in KCs is relevant in psoriasis.

Using a preclinical zebrafish larval model in combination with highly efficient CRISPR-Cas9-mediated editing and pharmacological manipulation, we observed that Gper1 deficiency aggravated skin alterations—namely, a higher number of KC aggregates and increased neutrophil infiltration—in a model of chronic skin inflammation. Conversely, treatment with the GPER1 agonist G-1 partially reversed the epithelial phenotype by reducing keratinocyte aggregates, although it did not affect neutrophil infiltration. Infiltration of neutrophils into the epidermis is one of the histological characteristics of psoriasis, and several studies suggest that neutrophils, monocytes, and macrophages facilitate the development of psoriasis. For example, psoriatic lesions have been reported to significantly improve during drug-induced agranulocytosis [17]. Estrogen has also been shown to modulate neutrophil functions, with some of these effects mediated by GPER1, a mechanism that is evolutionarily conserved [30, 31]. Nevertheless, our results show that the observed exacerbated neutrophil infiltration into the inflamed skin of Gper1-deficient larvae is not cell autonomous, since overexpression of Gper1 in neutrophils had no significant effects on skin alterations and neutrophil infiltration. Furthermore, skin inflammation per se, assayed as Nfkb and Il1b reporter activity, oxidative stress, and KC cell death were all unaffected by Gper1 deficiency, despite the ability of estrogens to downregulate IL-1β production in neutrophils and macrophages in a mouse psoriasis model via nuclear estrogen receptors [18], to suppress NF-κB in various cell populations in vitro [5], and to inhibit inflammation in different mouse models of immune-related diseases [11], to protect fetal health from maternal inflammation [32], and to protect cardiomyocytes from oxidative stress-induced death [33].

Although our study specifically focuses on GPER1, without addressing classical estrogen receptors or systemic estradiol effects due to toxicity in the zebrafish model, it is important to place our findings in the context of estrogen biology. The only known physiological activator of GPER1 is 17β-estradiol, which is known to exert both pro- and anti-inflammatory effects depending on context. A recent study showed that GPER1 expression is downregulated in human KC upon stimulation with psoriasis-related cytokines and suggested that GPER1 might mediate the protective effects of estradiol in this context [34]. These findings align with our own data showing reduced *GPER1* expression in psoriatic lesions and suggest that GPER1 signaling may represent a convergent anti-inflammatory mechanism downstream of estrogens. While we did not use estradiol due to its developmental toxicity in zebrafish [35], our genetic and pharmacological approaches demonstrate a specific role of Gper1 in controlling KC proliferation and immune cell infiltration, independently of broader estrogen effects. Further investigation of GPER1-mediated estrogen signaling in mammalian models of psoriasis (e.g., imiquimod-induced inflammation) will be important to fully understand its therapeutic potential.

One relevant observation of this study is that Gper1 decreased in a cell-autonomous manner the proliferation of KCs in the Spint1a-deficient model. This is not surprising, as GPER1 has been shown to reduce the proliferative capacity of melanoma cells [36] and engage in crosstalk with key growth factor receptors, such as the epidermal growth factor receptor (EGFR) and insulin-like growth factor-I receptor (IGF1R) [37]. Notably, EGFR appears to play a critical role in psoriasis [38]. Therefore, our results may have clinical relevance, as GPER1 signaling appears to regulate the uncontrolled proliferation of KC observed in psoriasis and atopic dermatitis— a pathological feature that represents an effective therapeutic target in these diseases [39]. Estrogen signaling in KCs seems to be very complex, since they express not only ERs but also the key steroidogenic enzymes required for estrogen biosynthesis. In fact, estrogens have been shown to stimulate KC proliferation through genomic and non-genomic activation of the extracellular signal-regulated kinases (ERK1 and ERK2) [40]. Furthermore, estrogens also stimulate epidermal and dermal cell migration and proliferation by enhancing the secretion of growth factors, such as EGF and transforming growth factor β (TGFβ), aiding re-epithelialization [41]. It seems, therefore, that estrogens acting through GPER1 show a protective role in the skin, promoting wound healing and preventing exacerbated inflammation.

Our study also suggests that KC proliferation is essential for enabling neutrophil infiltration into inflamed skin. This conclusion is supported not only by the effects of Gper1—whose loss promotes KC proliferation without directly altering inflammatory signaling—but also by direct pharmacological inhibition of cell division and by the proximity between infiltrated leukocytes and proliferating KC in the epidermis of psoriasis lesional skin. A similar mechanism has been described in Drosophila embryos, where macrophage invasion into ectodermal tissue requires epithelial cell division at the entry site to enable extracellular matrix disassembly [25]. That study also showed that the frequency of cell division influences invasion efficiency, although weakening cell adhesion can allow infiltration in a proliferation-independent manner. Whether such mechanisms are conserved in vertebrate skin remains to be elucidated. The facilitation of immune cell invasion into epithelial tissues by cell proliferation may have important clinical implications for psoriasis and other epithelial inflammatory diseases characterized by KC hyperproliferation. In these conditions, enhanced immune cell infiltration could further amplify inflammation, creating a self-perpetuating feedback loop between KC and immune cells.

In summary, our data highlight a previously unrecognized role of GPER1 in the regulation of skin inflammation, acting specifically through KC rather than immune cells. Although GPER1 has been implicated in immune regulation and inflammatory processes [10], its function in psoriasis remains largely unexplored. Our findings suggest that GPER1 is a promising therapeutic target for hyperproliferative inflammatory skin disorders. While no approved drugs currently target GPER1 directly, its classification as a G protein-coupled receptor (GPCR) makes it an attractive candidate for drug development, given that ~40% of FDA-approved drugs act on GPCRs. Importantly, targeting GPER1 may offer a safer alternative to conventional estrogen-based therapies, which are often associated with off-target effects such as increased risk of hormone-sensitive cancers due to systemic activation of ERα signaling. Pharmacological modulation of GPER1 could thus represent a novel and more selective approach to managing skin inflammatory diseases.

## Materials and methods

### Gene expression omnibus (GEO) database analysis on human skin samples

Human psoriasis transcriptomic data (accession number: GSD4602) were collected from the GEO database (https://www.ncbi.nlm.nih.gov/geo/). Gene expression plots were obtained using GraphPad Prism Software.

### Immunohistochemistry on human skin samples

Skin biopsies from healthy donors (*n* = 5) and psoriasis patients (*n* = 5) were used. Clinical inclusion criteria were a psoriasis diagnosis evaluated by 2 dermatologists independently and histology of skin lesions compatible with psoriasis/psoriasiform dermatitis evaluated by a pathologist. Clinical exclusion criteria were a diagnosis compatible with other dermatoses (atopic dermatitis, lichen planus, phytodermatosis and allergic contact eczema) and histology compatible with lichenoid dermatitis/lichen planus or spongiotic dermatitis. Sections were fixed in 4% PFA, embedded in Paraplast Plus and sectioned at a thickness of 5 μm. After being dewaxed and rehydrated, the sections were incubated in 10 mM citrate buffer (pH 6) at 95 °C for 20 min and then at room temperature for 15 min to retrieve the antigen. Next, steps were taken to block endogenous peroxidase activity and nonspecific binding. Afterward, sections were immunostained with rabbit polyclonal antibody to GPER1 (Sigma-Aldrich, #SAB2700363, 1/100), or rabbit monoclonal anti-pCREB (Cell Signaling Technology, #9198, 1/800), followed by 1/100 dilution of biotinylated anti-rabbit secondary antibody (Dako, E0432) and then by the ImmunoCruz goat ABC Staining System (#sc-2023, Santa Cruz Biotechnology). Finally, after adding DAB staining solution (Santa Cruz Biotechnology), the sections were dehydrated, rinsed and mounted in Neo-Mount (Sigma-Aldrich). Sections stained with DAB were finally examined under a Leica microscope equipped with a Leica DFC 280 digital camera. No staining was observed when the primary antibody was omitted.

For double immunofluorescent analysis of CD45 and PCNA in human skin biopsies, deparaffinized sections were rehydrated before performing antigen retrieval with Tris/EDTA buffer (10 mM Tris base, 1 mM EDTA solution, 0.05% Tween 20, pH 9.0). Then, they were blocked with 5% BSA in PBS for 30 mins at room temperature, washed with PBS and immunostained with a rabbit anti-human CD45, clone 5L3 ZooMAb, antibody (1:100, Sigma-Aldrich, #ZRB1180) and a mouse anti-PCNA antibody (1:500, Sigma-Aldrich, #P8825), followed by the secondary antibodies donkey anti-rabbit IgG H&L Alexa Fluor® 488 (Thermo Fisher, #A21206) and goat anti-mouse IgG H&L Alexa Fluor® 594 (Thermo Fisher, #A11032), both at 1:400, in incubation buffer (PBS + 1% BSA + 0,3% triton X-100). Sections were then washed with PBS and incubated with DAPI (1:5000; Sigma Aldrich #32670), 5–7 min at darkness at room temperature. Finally, they were mounted using VECTASHIELD® Antifade Mounting Medium with DAPI (Vector Laboratories, #H-1200) and examined under a confocal microscope, Leica STELLARIS 8 and further processed with Leica software and ImageJ. No staining was observed when primary antibodies were omitted. The objective used was x63. Analysis of the nearest neighbor assessing the Shortest Distance to Surface by Imaris; from surface 1 (green; CD45) to surface 2 (red; PCNA).

### Zebrafish lines

Zebrafish (*Danio rerio* H.) were obtained from the Zebrafish International Resource Center and were mated, staged, raised and processed as described [42]. The lines *Tg(lyz:DsRED2)*^*nz50*^ [43], *Tg(6xNF-KB:eGFP)*^*sh235*^ (*Tg(nfkb:eGFP)* for simplicity) [44]*, Tg(il1b:GFP-F)*^*ump3*^ [45], *Tg(mpx:Gal4.VP16)*^*i222*^ [46], *TgBAC(ΔNp63:Gal4)*^*la213*^ [47] have been previously described. *spint1a*^*hi2217Tg/hi2217Tg*^ was isolated from insertional mutagenesis screens [48]. *Tg(uas:gper1)*^*ums8*^ was generated by microinjecting a solution containing 100 ng/μl *uas:gper1* construct and 50 ng/μl Tol2 RNA into the yolk sac of one-cell-stage embryos using a microinjector (Narishige). The *uas:gper1* construct was generated using the Tol2 kit and harbors a GFP driven by the heart-specific promoter *myl7* for rapid screening.

### Genetic inhibition in zebrafish

CRISPR RNA (crRNA) obtained from Integrated DNA Technologies (IDT) with the following target sequence were used: *gper1* crRNA: 5′- AGATCTGTACTTTGTCAACC-3′, *spint1a* crRNA: 5′- TACGAATGGGAGCAAGTGGT -3′. A negative control crRNA (catalog no. 1072544, crSTD) was also acquired from IDT. They were resuspended in nuclease-free duplex buffer to 100 µM. One µl of each crRNA was mixed with 1 µl of tracrRNA (trans-activating tracrRNA; catalog no. 1072533) and incubated at 95 °C for 5 min for duplexing. Next, the duplex mix (gRNA: crRNA + tracrRNA) was cooled for 5 min at room temperature, and it was diluted to 1000 ng/µl by adding 1.43 µl of nuclease-free duplex buffer. The injection mix was prepared by mixing 1 µl of gRNA, 0.2 µl of Cas9 nuclease V3 (IDT, catalog no. 1081058), 2.55 µl of nuclease-free duplex buffer and 0.5 µl of phenol red in the case of *spint1a* crRNA or 2 µl of gRNA, 0.4 of Cas9 nuclease, 1.35 µl of nuclease-free duplex buffer and 0.5 µl of phenol red in the case of gper1 gRNA. This mix was microinjected into the yolk sac of one-cell-stage embryos using a microinjector (Narishige). The editing efficiency of *gper1* crRNA was determined by amplification of the target sequence with a specific pair of primers (F: 5’-TAAAACAGAGACCTGGTCAGCA-3’, R: 5’-TTGTCGACGGATTTGGGTTATT-3’) and analysis by the TIDE webtool (https://tide.nki.nl/) [49].

### Chemical treatment in zebrafish

Zebrafish embryos were manually dechorionated at 24 h post-fertilization (hpf). Larvae were treated from 24 hpf to 72 hpf with either the CDK4/CDK6 inhibitor Palbociclib (10 µM, #HY-50767, MCE) or with the GPER1 agonist G-1 (1, 0.5, 0.1 and 0.01 µM, #415919-74-3, Sigma-Aldrich) by bath immersion at 28 °C. Incubation was carried out in 6-well plates containing 15 to 20 larvae/well in egg water supplemented with 1% dimethyl sulfoxide (DMSO) to facilitate drug absorption. Control larvae were incubated only with DMSO.

### Imaging of zebrafish larvae

Live imaging of 3-day post-fertilization (dpf) larvae was obtained after anesthetizing them with buffered tricaine dissolved in egg water. The images were acquired with the integrated camera on an epifluorescence LEICA MZ16FA stereomicroscope set up with green and red fluorescent filters. The number of KC aggregates, which are readily visible under bright-field microscopy as dense clusters of keratinocytes in the outer layers of the skin, and the number and distribution of neutrophils (lyz^+^ cells) were determined by counting them in blind samples [21]. To estimate neutrophil infiltration into the skin, we primarily quantified the percentage of neutrophils located outside the CHT. While in wild-type larvae the vast majority of neutrophils (~90%) remained within the CHT at 2–3 dpf, in Spint1a-deficient larvae approximately 40% were found outside this region, indicating active skin infiltration [21]. H_2_O_2_ release was determined employing the live cell fluorogenic substrate acetyl-pentafluorobenzene sulphonyl fluorescein (Cayman Chemical) [50]. Briefly, 20 embryos of 3 dpf were collected in a well of a 24-well plate with 50 μM of the substrate in 1% DMSO for 1 h. H_2_O_2_ production was quantified by measuring mean intensity fluorescence in skin with ImageJ (FIJI) software. This quantification method was also used to determine Nfkb and Il1b reporter activity using *Tg(nfkb:eGFP)* and *Tg(il1b: GFP)* lines, respectively.

### TUNEL assay

TUNEL assay was performed to assess the number of apoptotic cells in 2 dpf embryos. Specimens were fixed in 4% paraformaldehyde for 2 h, washed three times with PBS for 3 min each, dehydrated in graded methanol: PBST (PBS + 0.1% Tween 20) series (1:3, 1:1, 3:1) for 5 min each and then transferred to methanol at –20 °C. The next day, samples were rehydrated in graded methanol: PBST series (3:1, 1:1, 1:3) for 5 min each and finally washed in PBS for 5 min. Afterward, samples were rinsed with precooled (−20 °C) 100% acetone and then incubated in 100% acetone at −20 °C for 10 min. Subsequently, embryos were washed 3 times for 10 min with PBST and incubated in a permeabilization solution (0.1% TritonX-100 and 0.1% sodium citrate in PBS) for 30 min. Samples were then washed twice for 5 min in PBST and incubated with 50 μL fresh TUNEL reaction mixture, consisting of 5 μL of enzyme solution mixed with 45 μL of labeling solution (In Situ Cell Death Detection Kit, Fluorescein, Roche), at 37 °C for 1 h. Finally, they were washed five times with PBST for 5 min each. As a TUNEL positive control, 2-dpf zebrafish embryos treated with DNAse I Solution (ThermoFisher Scientific #89836, 20 U/ml) in DNAse I buffer, incubated at room temperature for 10 min were used. Images were acquired on an epifluorescence LEICA MZ16FA stereomicroscope set up with green and red fluorescent filters, and the number of apoptotic cells was determined by counting them in blind samples.

### BrdU proliferation assay

5-Bromo-2’-deoxyuridine (BrdU) incorporation was used to determine the number of proliferating cells in embryos at the age of 2 dpf. They were dechorionated and cooled for 15 min on ice in egg water. Subsequently, they were placed in 10 mM BrdU solution with 15% DMSO and incubated 20 min on ice to allow uptake of BrdU. Next, BrdU solution was removed, and samples were washed in egg water at 28.5 °C for 5 min. Immediately after that, larvae were fixed and dehydrated as described above. On the following day, samples were gradually rehydrated and finally washed twice in PBST for 5 min. Embryos were incubated in acetone at –20 °C for 10 min as described above. After three quick washes in water and two washes in 2M HCl, larvae were incubated in 2M HCl for 1 h to denature the labeled DNA and expose the BrdU epitope. Next, samples were neutralized with 0.1 M borate buffer (100 mM sodium tetraborate, 100 mM boric acid), pH 8.5, for 20 min at room temperature. Larvae were washed 5 times for 5 min each with PBST and incubated in blocking solution (10% fetal bovine serum, 1% DMSO, 0.1% Tween 20 in PBS) for 1 h. Following, they were incubated in mouse monoclonal anti-BrdU antibody (Sigma-Aldrich, B8434, 1:200) in BrdU blocking solution overnight at 4 °C. The next day, samples were washed five times for 10 min with PBST, incubated with fluorophore-conjugated anti-mouse secondary antibody Alexa Fluor 488 (Invitrogen, #A28175, 1:1000) for 2 h and again washed five times for 10 min with PBST. Finally, images were captured on an epifluorescence LEICA MZ16FA stereomicroscope, and the number of proliferating cells was determined by counting them in blind samples.

### Whole larvae pH3 immunofluorescence

Embryos at 2 dpf were fixed in 2% paraformaldehyde overnight at 4 °C. The next day, samples were washed 3 times with PBS for 3 min each and incubated in blocking solution (PBS + 0.3% Triton X-100 + 4% BSA) for 1 h at 4 °C. Following, they were incubated in anti-Phospho-Histone H3 (pH3) antibody (Cell Signaling, #9701, 1:100) diluted in blocking solution for two days at 4 °C. Subsequently, samples were washed 3 times with PBT (PBS + 0.3% Triton X-100) for 5 min each and incubated with anti-rabbit secondary antibody Alexa Fluor 488 (Invitrogen, #A11008, 1:500) diluted in blocking solution for 2 h at room temperature. Finally, embryos were washed three times with PBT for 5 min each and images were captured on an epifluorescence LEICA MZ16FA stereomicroscope, and the number of proliferating cells was determined by counting them in blind samples.

### Statistical analysis

Sample sizes were based on the authors’ experience with molecular and in vivo studies, as published in multiple previous reports. For animal models, experiments were designed to detect differences between treatment groups or genotype-dependent effects with 80% power at *p* = 0.05. Embryos were randomly allocated to each experimental condition and analyzed in blind samples. No data were excluded from the analysis. Data are shown as mean ± S.E.M. The differences between the two samples were analyzed by the two-sided Student’s *t* test. The data met the normal distribution assumption. In cases with three or more conditions, statistics were carried out by analysis of variance (ANOVA) and a Tukey multiple range test or the non-parametric Kruskal-Wallis test, as appropriate. Whelch’s correction was performed when there were unequal variances. To analyze the relation between two variants, Linear regression and the Pearson correlation test were performed. A *p*-value of <0.05 was considered statistically significant. Statistical analysis was performed using Prism 8.0 (GraphPad Software, CA, USA).

## Data Availability

The datasets generated and/or analyzed during the current study are available from the corresponding author (VM) on reasonable request.
